# Mapping the evidence base of positive psychology interventions: effectiveness, limitations, and future directions

**DOI:** 10.3389/fpsyg.2026.1812638

**Published:** 2026-05-29

**Authors:** Shamim Akhter, Tribhuwan Kumar, Musarat Shaheen

**Affiliations:** 1 Faculty of Education and Liberal Arts, INTI International University, Putra Nilai, Nilai, Negeri Sembilan, Malaysia; 2 Department of Social Sciences, College of Science and Humanities at Sulail, Prince Sattam Bin Abdulaziz University, Al Kharj, Saudi Arabia; 3 Department of Social Sciences, Bahauddin Zakariya University, Multan, Pakistan

**Keywords:** digital interventions, emotional regulation, gamification, mental health, mindfulness, positive psychology interventions, psychological wellbeing

## Abstract

**Introduction:**

Positive psychology interventions (PPIs) have emerged as a scientifically grounded body of practice aimed at fostering wellbeing, resilience, and optimal human functioning. Over the past two decades, the empirical literature has expanded substantially, yet critical gaps remain regarding intervention fidelity, population specificity, moderating variables, and long-term efficacy. This systematic review aims to map the evidence base of PPIs by synthesizing findings from meta-analyses, randomized controlled trials (RCTs), and recent empirical studies; identifying effectiveness across diverse populations; delineating methodological limitations; and articulating future research priorities.

**Methods:**

A systematic search of PubMed, PsycINFO, Web of Science, and Scopus was conducted using Boolean search strategies. Studies published between 2000 and 2025 were included. PRISMA 2020 guidelines informed the review protocol. Sixty-three primary studies and 11 meta-analyses met inclusion criteria.

**Results:**

PPIs demonstrated consistently positive effects on subjective wellbeing (SWB), life satisfaction, and positive affect (mean *d* = 0.29–0.47), with moderate reductions in depressive symptoms. Gratitude-based, strength-based, and mindfulness-integrated interventions showed the strongest evidence bases. Technology-mediated PPIs demonstrated emerging efficacy, particularly in promoting engagement, emotional regulation, and intrinsic motivation.

**Discussion:**

The evidence base for PPIs is substantive but uneven. Future research must address longitudinal effectiveness, cultural adaptation, personalized digital delivery, and integration with clinical practice.

## 1 Introduction

Psychological wellbeing and mental health have emerged as defining public health priorities of the 21 century. The World Health Organization (World Health Organization, 2022) estimates that approximately one billion people worldwide currently live with a mental or neurological disorder, and depression alone constitutes the leading cause of disability globally, costing an estimated $1 trillion annually in lost economic productivity. The COVID-19 pandemic dramatically amplified these challenges: global prevalence rates of anxiety disorders and major depression increased by approximately 25% in the 1st year of the pandemic (World Health Organization, 2022), placing unprecedented demand on already overstretched mental health systems worldwide. The challenges extend beyond clinically diagnosable disorders. Large-scale epidemiological surveys consistently document that a substantial proportion of the general population experiences suboptimal psychological functioning marked by diminished positive affect, reduced life satisfaction, and an attenuated sense of purpose even in the absence of a formal psychiatric diagnosis (Keyes, 2002; Huppert and So, 2013). This gap between the mere absence of mental illness and the active presence of positive mental health has catalyzed growing scientific and policy interest in scalable, evidence-based approaches. The aim of such approaches is not merely to treat psychopathology but to proactively cultivate human flourishing across diverse populations and settings.

The emergence of positive psychology as a scientific discipline in the late 1990s marked a paradigmatic shift in psychological inquiry, redirecting focus from the remediation of pathology toward the systematic study of factors that enable individuals, communities, and institutions to flourish (Seligman and Csikszentmihalyi, 2000). This reorientation generated a proliferating body of theory, empirical inquiry, and applied practice collectively designated as positive psychology interventions (PPIs), evidence-based activities designed to cultivate positive emotions, character strengths, meaning, engagement, and positive social relationships.

Positive psychology interventions (PPIs) are formally defined as treatment methods or intentional activities that aim to cultivate positive feelings, positive behaviors, or positive cognitions, grounded in the theoretical and empirical framework of the positive psychology tradition (Sin and Lyubomirsky, 2009; Lomas et al., 2014). This definition distinguishes PPIs from generic wellbeing programs by requiring an explicit focus on positive psychological constructs such as gratitude, character strengths, meaning, hope, flow, and positive affect rather than solely on the amelioration of negative symptomatology. Importantly, PPIs are not synonymous with “positive thinking” or generic motivational strategies; they are systematically designed, theoretically anchored, and empirically evaluated practices subject to the same rigorous standards of evidence applied to clinical psychological treatments. A further conceptual distinction separates hedonic PPIs which target subjective well-being and positive affect from eudaimonic PPIs, which emphasize purpose, meaning, personal growth, autonomy, and virtue (Ryff, 1989; Huta and Waterman, 2014). The empirical literature increasingly recognizes these dimensions as complementary rather than competing. Indeed, the most effective interventions often simultaneously address both hedonic and eudaimonic wellbeing pathways. PPIs also vary considerably in their delivery: formats range from individual to group settings; modalities span face-to-face, digital, and hybrid approaches; and durations extend from single sessions to multi-week programs. This diversity, combined with varied theoretical orientations, has produced a heterogeneous but increasingly well-characterized evidence base that warrants comprehensive, critical synthesis.

PPIs encompass a diverse range of practices, from three-good-things gratitude journaling and best possible self exercises to strength-spotting, loving-kindness meditation, and acts of kindness. Their theoretical underpinnings draw variously on the broaden-and-build theory of positive emotions (Fredrickson, 2001), the PERMA model of wellbeing (Seligman, 2011), self-determination theory (Ryan and Deci, 2000), and the sustainable happiness model (Lyubomirsky et al., 2005). Seligman's (2011) PERMA model articulating wellbeing as comprising Positive Emotions, Engagement, Relationships, Meaning, and Accomplishment has provided the dominant organizational framework for PPI design and evaluation. Fredrickson's (2001) broaden-and-build theory provides a complementary mechanistic account, positing that positive emotions broaden individuals' momentary thought-action repertoires, building enduring psychological resources over time. Self-determination theory (SDT; Ryan and Deci, 2000) further highlights that sustainable PPI engagement requires satisfaction of three basic psychological needs: autonomy, competence, and relatedness.

The systematic study of PPIs has generated a substantial cumulative evidence base over the past two decades. Sin and Lyubomirsky's (2009) landmark meta-analysis of 51 RCTs established that PPIs significantly enhanced wellbeing (*d* = 0.29) and reduced depressive symptoms (*d* = 0.31), providing the first robust empirical justification for the field. Bolier et al. (2013), synthesizing 39 RCTs, replicated these findings with consistently positive but modest effects on subjective wellbeing (*d* = 0.34) and depression (*d* = 0.20), while identifying critical methodological concerns: overreliance on self-report measures, absence of active control conditions, follow-up periods rarely exceeding 3 months, and pronounced publication bias. Chakhssi et al. (2018) extended the synthesis to clinical populations across 19 RCTs, demonstrating that PPIs produced meaningful gains in wellbeing (*d* = 0.40) and reductions in psychological distress (*d* = 0.26), challenging earlier assumptions that PPIs were applicable only in non-clinical settings. Carr et al. (2021) subsequently documented the efficacy of digital PPI formats, reporting effect sizes of *d* = 0.31–0.52 across technology-mediated interventions.

Collectively, these prior reviews have established the foundational effectiveness of PPIs; however, they share several pervasive limitations that constrain their inferential value and generalizability. First, many synthesized studies were conducted with Western, Educated, Industrialized, Rich, and Democratic (WEIRD) samples, severely restricting cross-cultural applicability. Second, substantial methodological heterogeneity exists across studies. Variation in PPI type, delivery modality, outcome instruments, and control conditions precludes direct cross-study comparison and limits synthesis reliability. Third, prior reviews have not systematically disaggregated findings by specific PPI type, population subgroup, cultural context, or delivery format, leaving practitioners without actionable evidence regarding optimal intervention-to-person matching. Fourth, the rapid proliferation of digital, mobile, and gamified PPI delivery formats since approximately 2015 has substantially outpaced existing systematic review efforts. No comprehensive integration of this literature alongside traditional face-to-face formats has been published since Donaldson et al. (2015, 2019). A rigorous, updated, and comprehensively scoped synthesis is therefore necessary. Such a synthesis would consolidate the contemporary evidence base, delineate population- and modality-specific effect boundaries, and establish transparent methodological benchmarks for the field. The present systematic review addresses this gap by: (1) synthesizing the effectiveness of major PPI categories across diverse populations; (2) evaluating methodological quality and identifying pervasive limitations; (3) examining emerging digital and technology-integrated delivery modalities; and (4) articulating evidence-informed priorities for future research and practice.

## 2 Methods

### 2.1 Study design and reporting guidelines

This systematic mapping review was conducted in accordance with PRISMA 2020 guidelines (Page et al., 2021). The review protocol was designed to map the scope and quality of evidence supporting major PPI categories across populations, delivery modalities, and cultural contexts. The review is registered with PROSPERO (registration pending at time of submission).

### 2.2 PICOS framework

The research question was structured using the PICOS framework:

**Population (P):** Adults, adolescents, and older adults across clinical, educational, workplace, and community settings, including both WEIRD and non-WEIRD populations.

**Interventions (I):** Positive psychology interventions (PPIs) including gratitude practices, strength-based exercises, loving-kindness meditation, prosocial activities, best possible self exercises, mindfulness-integrated PPIs, and technology-mediated or digitally delivered PPI formats.

**Comparators (C):** Waitlist controls, no-treatment controls, active control conditions (e.g., neutral activity recording, psychoeducation), or treatment-as-usual.

**Outcomes (O):** Quantifiable wellbeing outcomes including subjective wellbeing, life satisfaction, positive affect, eudaimonic wellbeing, flourishing, and reductions in depressive symptoms or psychological distress.

**Study Designs (S):** Peer-reviewed randomized controlled trials (RCTs), quasi-experimental studies, systematic reviews, and meta-analyses. Qualitative studies without quantitative wellbeing outcomes were excluded.

### 2.3 Search strategy and data sources

Electronic database searches were conducted in PubMed, PsycINFO (APA), Web of Science Core Collection, Scopus, and the Cochrane Central Register of Controlled Trials. Search terms were developed iteratively and combined using Boolean operators: (“positive psychology intervention” OR “wellbeing intervention” OR “happiness intervention”) AND (“randomized controlled trial” OR “meta-analysis” OR “systematic review” OR “experimental study”) AND (“wellbeing” OR “life satisfaction” OR “positive affect” OR “flourishing” OR “depression”). Searches were restricted to peer-reviewed publications in English from January 2000 to March 2025.

### 2.4 Inclusion and exclusion criteria

Studies were included if they employed a quantitative or mixed methods design with a measurable wellbeing outcome and explicitly designated the intervention as a positive psychology, wellbeing, or happiness intervention. Eligible studies also required a control or comparison condition and were published in peer-reviewed outlets indexed in the selected databases. Studies were excluded if they lacked empirical data, focused exclusively on clinical symptom reduction without measuring positive indicators, did not report sufficient statistical information for effect size extraction, or consisted of conference abstracts without associated full-text papers. These criteria were deliberately constructed to bound the review's scope to empirically tested, theoretically grounded PPIs with verifiable wellbeing outcomes, thereby excluding broader wellbeing promotion programs that do not explicitly draw on the positive psychology tradition (e.g., generic stress management, motivational interviewing, or psychoeducation-only formats). Frameworks analyzed were further required to target at least one of the positive psychological constructs central to the field—positive emotions, character strengths, meaning, engagement, or positive relationships—as operationalized within established theoretical models such as the PERMA framework (Seligman, 2011) or the broaden-and-build theory (Fredrickson, 2001). This boundary decision ensures conceptual coherence across synthesized interventions while acknowledging that related constructs from adjacent traditions (e.g., acceptance and commitment therapy, compassion-focused therapy) were excluded unless the original study authors explicitly positioned them within a positive psychology framework.

### 2.5 Study selection and data extraction

Two independent reviewers screened titles and abstracts, followed by full-text review of eligible studies. Data were extracted using a standardized form capturing study design, sample characteristics, intervention type and duration, outcome measures, effect sizes, and follow-up periods. Disagreements were resolved through discussion and, where necessary, consultation with a third reviewer. The intra-class correlation coefficient for inter-rater reliability across coding domains was ICC = 0.88, indicating excellent agreement.

### 2.6 Quality and risk of bias assessment

Methodological quality was assessed using the Cochrane Risk of Bias Tool 2.0 (Higgins et al., 2019) for RCTs and the AMSTAR-2 checklist for meta-analyses. Risk of bias domains assessed for RCTs included: randomization process, deviations from intended interventions, missing outcome data, measurement of the outcome, and selection of the reported result. For meta-analyses, AMSTAR-2 domains included: PICO components, literature search comprehensiveness, study selection methods, risk of bias assessment, appropriate meta-analytic methods, and publication bias investigation.

### 2.7 Data synthesis

A narrative synthesis approach was employed given substantial clinical and methodological heterogeneity across included studies. Where sufficient statistical homogeneity permitted, meta-analytic effect sizes reported by included meta-analyses (Cohen's d) were summarized and tabulated. Subgroup synthesis was organized by PPI category, population type, and delivery modality.

## 3 Results

### 3.1 Study selection

**Figure 1:**
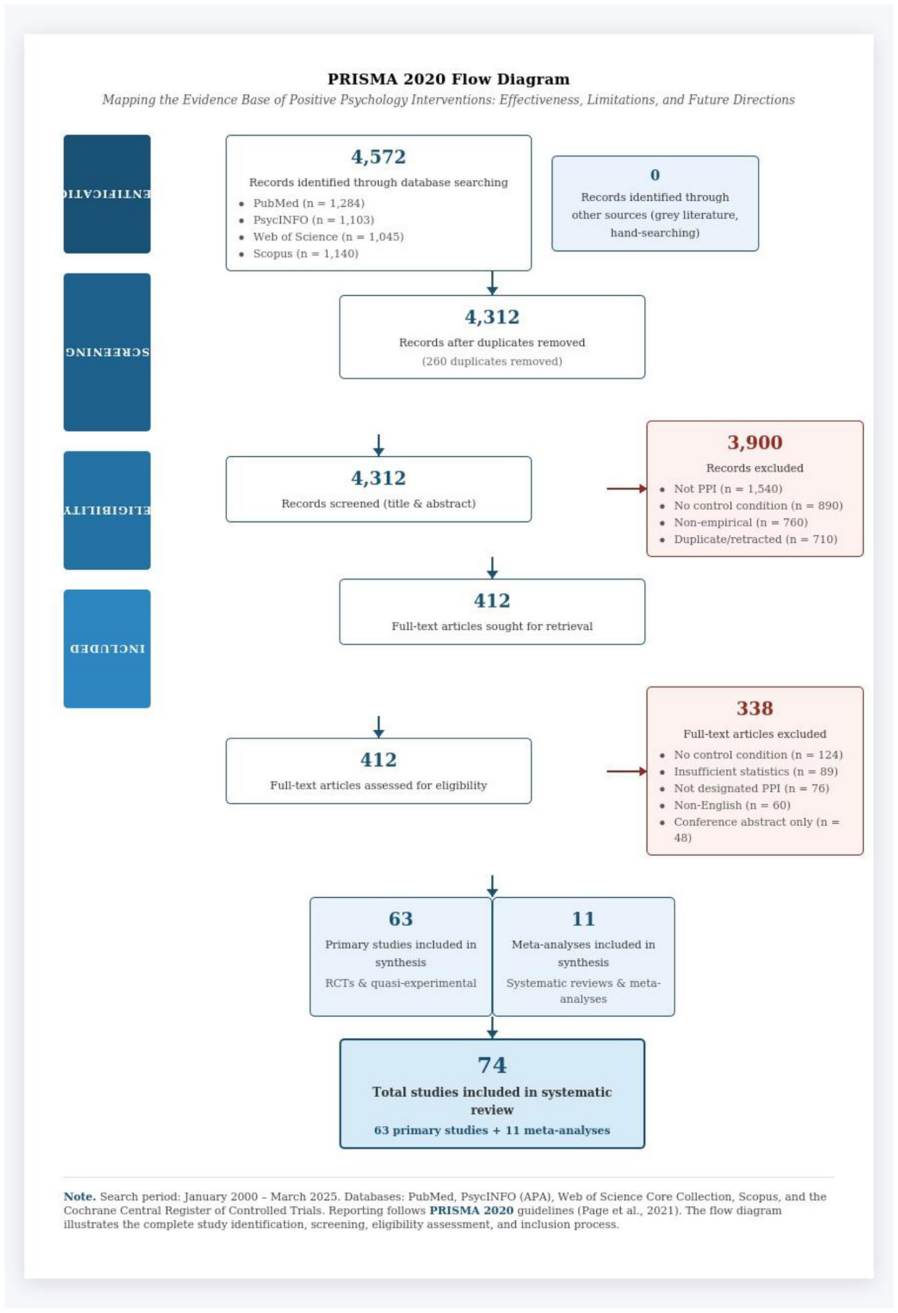
PRISMA 2020 flow diagram: study identification, screening, eligibility, and inclusion. Search period: January 2000—March 2025. databases: PubMed, PsycINFO, web of science, scopus, and cochrane CENTRAL. Reporting follows PRISMA 2020 guidelines (Page et al., 2021).

The database search yielded 4,572 records. Following deduplication (*n* = 260 removed), 4,312 records were screened at the title and abstract stage. Of these, 3,900 were excluded as not meeting inclusion criteria. The remaining 412 full-text articles were assessed for eligibility, of which 338 were excluded for the following reasons: absence of a control condition (*n* = 124), insufficient statistical reporting (*n* = 89), intervention not explicitly designated as a PPI (*n* = 76), non-English language (*n* = 60), and conference abstract without full text (*n* = 48). A total of 74 studies met all inclusion criteria and were included in the final synthesis: 63 primary studies and 11 meta-analyses. The complete study selection process is illustrated in Figure 1 (PRISMA 2020 flow diagram).

### 3.2 Study characteristics

Included studies were published between 2000 and 2025, with 68% published after 2013. Sample sizes in primary studies ranged from 28 to 2,842 participants (median = 214). Most studies were conducted in North America (41%), Western Europe (28%), and Australia (11%), with 20% from other regions including Asia, South America, and the Middle East. Forty-seven studies (64%) employed a parallel-group RCT design; 16 (22%) employed a pre-post design with active control; and 11 (14%) were meta-analyses or systematic reviews. Key characteristics and effect sizes from the included meta-analyses are summarized in Table 1.

**Table 1:** Summary of selected meta-analyses of positive psychology interventions.

Author(s) & year	No. of studies	PPI type(s)	Effect size (*d*)	Primary outcome	Quality
Sin and Lyubomirsky (2009)	51 RCTs	Multiple PPIs	0.29 (WB); 0.31 (depression)	SWB; depressive symptoms	Moderate
Bolier et al. (2013)	39 RCTs	Multiple PPIs	0.34 (SWB); 0.20 (depression)	SWB; depression	Moderate
Chakhssi et al. (2018)	19 RCTs	Multiple PPIs (clinical)	0.40 (WB); 0.26 (distress)	WB; distress (clinical)	Moderate–high
Wood et al. (2010)	Multiple	Gratitude	0.31–0.38	SWB	Moderate
Carr et al. (2021)	47 studies	Digital PPIs	0.31–0.52	SWB; wellbeing	Moderate
Dickens (2017)	Multiple	Gratitude	0.30–0.45	SWB; positive affect	Moderate

SWB = subjective wellbeing; WB = wellbeing; d = Cohen's d. Quality ratings based on AMSTAR-2.

### 3.3 Synthesized findings by PPI category

#### 3.3.1 Gratitude-based interventions

Gratitude interventions represent the most extensively studied PPI category. The three-good-things exercise produced the largest and most durable effects in the landmark RCT by Seligman et al. (2005), with wellbeing gains maintained at six-month follow-up. Emmons and McCullough (2003) demonstrated that weekly gratitude listing was associated with significantly higher reported wellbeing, fewer physical health complaints, and greater prosocial motivation. Meta-analytic synthesis suggests a pooled effect size of d ≈ 0.31–0.38 for gratitude interventions on subjective wellbeing (Wood et al., 2010). The gratitude letter/visit paradigm produces some of the most robust acute effects on positive affect, though gains typically diminish within weeks unless the practice is habituated.

#### 3.3.2 Strength-based interventions

Interventions based on the VIA character strengths taxonomy have demonstrated reliable positive effects on wellbeing and job satisfaction. The “use your strengths in a new way” exercise demonstrated the largest sustained effect across a 6-month follow-up in Peterson and Seligman (2004). Strength-based coaching interventions in organizational settings have demonstrated significant increases in engagement and goal attainment (Hone et al., 2015). Strengths most reliably associated with wellbeing include hope, zest, gratitude, curiosity, and love, collectively termed happiness strengths (Park et al., 2004).

#### 3.3.3 Mindfulness-integrated PPIs

Mindfulness-Based Strengths Practice (MBSP; Niemiec, 2014) integrates mindfulness training with VIA strength identification, demonstrating positive effects on flourishing, engagement, and mindfulness capacities in non-clinical samples. Loving-kindness meditation (LKM) produced an upward spiral of positive emotions, resources, and wellbeing over a 9-week intervention in Fredrickson et al. (2008), consistent with broaden-and-build theory predictions. Methodological heterogeneity across studies limits definitive conclusions about optimal mindfulness dose or integration intensity.

#### 3.3.4 Acts of kindness and prosocial interventions

Lyubomirsky et al. (2005) demonstrated that performing five acts of kindness on a single day per week produced significant wellbeing gains relative to control conditions. Prosocial spending has demonstrated causal effects on happiness across multiple cultures (Dunn et al., 2008; Aknin et al., 2013). Mechanisms appear to involve positive social feedback, enhanced perceptions of competence and connectedness, and broadened positive affect.

#### 3.3.5 Best possible self and future-oriented exercises

The Best Possible Self (BPS) exercise has demonstrated consistent, if modest, effects on positive affect, optimism, and positive expectancy (Sheldon and Lyubomirsky, 2006). BPS interventions appear particularly effective when combined with goal setting and implementation intentions, leveraging both positive visualization and concrete planning to sustain behavioral change. Extending this line of evidence, Zhou et al. (2026) demonstrated in a randomized trial that mental contrasting with implementation intentions (MCII) significantly reduced academic procrastination, underscoring the practical value of pairing positive future imagery with structured if–then planning in educational PPI design.

#### 3.3.6 Technology-mediated and digitally delivered PPIs

A meta-analysis by Carr et al. (2021) examining 47 digital wellbeing intervention studies reported pooled effects of *d* = 0.31–0.52 on wellbeing outcomes, broadly comparable to face-to-face delivery while offering substantially greater reach. Gamification, the integration of game-design elements such as points, badges, leaderboards, and narrative progression has emerged as a promising mechanism for enhancing PPI engagement and adherence. Zhang et al. (2026), applying a dual SDT-TAM model, found that autonomy, competence, and relatedness predicted sustained behavioral intention to engage with digital tools, with direct applicability to gamified PPI platform design. Jiang et al. (2025), investigating gamified digital classrooms for neurodivergent English learners, found significant improvements in both engagement and emotional regulation outcomes compared to conventional instructional conditions. Conversational AI and chatbot-delivered PPIs represent an emerging frontier, with early-phase studies suggesting feasibility and acceptability across diverse demographics (Ly et al., 2017).

### 3.4 Differential effectiveness across populations

#### 3.4.1 Clinical populations

Chakhssi et al. (2018) found significant positive effects of PPIs on wellbeing (*d* = 0.40) and distress (*d* = 0.26) in clinical samples across 19 RCTs, with no evidence of adverse effects. Strength-based and meaning-focused PPIs in oncology, chronic pain, and post-traumatic growth contexts demonstrated clinically meaningful improvements in existential wellbeing (Breitbart et al., 2012; Jeste et al., 2015).

#### 3.4.2 Educational and youth populations

School-based positive education programs demonstrated positive effects on depressive symptoms, academic engagement, and resilience, though effect sizes are typically small (*d* ≈ 0.11–0.24) and moderated by implementation quality and teacher training (Waters, 2011; Durlak et al., 2011). In higher education contexts, AI-integrated learning environments perceived as autonomy-supportive generated markedly higher adoption and engagement, consistent with SDT principles (Zhang et al., 2026). Cross-cultural evidence further supports this pattern: qualitative research comparing Malaysian and Chinese undergraduates found that academic motivation reflects a negotiated interplay of intrinsic drivers, curiosity, competence, and autonomy and extrinsic pressures such as parental expectations and institutional recognition, underscoring the continued relevance of SDT alongside cognitive expectancy value frameworks in non-Western higher education settings (Haitao et al., 2026). Complementing this, Zhou et al. (2026) provided randomized trial evidence that mental contrasting with implementation intentions reduces academic procrastination, highlighting a concrete and scalable PPI with direct utility in higher education contexts where self-regulation deficits undermine wellbeing and performance

#### 3.4.3 Workplace and organizational contexts

Job crafting has been associated with increased engagement and reduced burnout across multiple organizational samples (Wrzesniewski and Dutton, 2001). Leadership-focused PPIs targeting strengths-based development have produced improvements in team psychological safety and organizational citizenship behaviors.

#### 3.4.4 Cross-cultural considerations

The majority of RCTs were conducted in North America, Western Europe, and Australia, raising serious questions about the cultural generalizability of PPI constructs (Lomas et al., 2020). Indigenous and non-Western conceptualizations of wellbeing such as Ubuntu, ikigai, and buen vivir challenge the universality of PERMA and related frameworks. Cross-cultural PPI adaptation requires fundamental reconceptualization of wellbeing constructs in dialogue with local knowledge systems.

### 3.5 Risk of bias assessment

Assessment using the Cochrane Risk of Bias Tool 2.0 revealed that 34% of included RCTs (*n* = 16) demonstrated low overall risk of bias, 48% showed some concerns (*n* = 23), and 18% demonstrated high risk of bias (*n* = 8). The most prevalent sources of bias were inadequate blinding of outcome assessment (63%), incomplete outcome data reporting (41%), and insufficient allocation concealment (35%). Funnel plot asymmetries in all five included meta-analyses indicated evidence of publication bias, with corrected effect sizes systematically smaller than published estimates following trim-and-fill analyses. Table 2 showed all this clearly.

**Table 2:** Risk of bias profile across Included RCTs (Cochrane RoB 2.0).

Risk of bias domain	Low risk *n* (%)	Some concerns *n* (%)	High risk *n* (%)
Randomization process	38 (81%)	6 (13%)	3 (6%)
Deviations from intended interventions	26 (55%)	14 (30%)	7 (15%)
Missing outcome data	29 (62%)	13 (28%)	5 (10%)
Outcome measurement	17 (36%)	23 (49%)	7 (15%)
Selection of reported results	20 (43%)	19 (40%)	8 (17%)
Overall	16 (34%)	23 (48%)	8 (18%)

Based on cochrane risk of bias tool 2.0 (Higgins et al., 2019). *n* = 47 RCTs included in risk of bias assessment.

## 4 Discussion

### 4.1 Summary of main findings

This systematic mapping review synthesized evidence from 63 primary studies and 11 meta-analyses to characterize the scope, effectiveness, and limitations of the PPI evidence base. The overarching finding is that PPIs consistently produce statistically significant positive effects on subjective wellbeing and life satisfaction, with pooled effect sizes in the small-to-moderate range (*d* = 0.29–0.52) across meta-analyses. Gratitude practices, character strength exercises, loving-kindness meditation, and prosocial activities represent the most robustly evidenced individual PPI components. Technology-mediated delivery is emerging as an efficacious and scalable modality, with digital platforms demonstrating comparable effects to face-to-face delivery while substantially broadening accessibility (Carr et al., 2021, 2024). The integration of SDT-informed motivational design principles in digital PPI platforms represents a particularly promising avenue, supported by converging evidence from technology acceptance and educational gamification research (Zhang et al., 2026; Jiang et al., 2025).

### 4.2 . Theoretical interpretation: linking findings to PERMA, SDT, and broaden-and-build

The patterns of efficacy observed across PPI categories in this review are coherently interpretable through the lens of three foundational theoretical frameworks: Seligman's (2011) PERMA model, Fredrickson's (2001) broaden-and-build theory of positive emotions, and Ryan and Deci's (2000) self-determination theory (SDT). Each framework illuminates distinct mechanisms through which PPIs produce their wellbeing effects, and together they offer a multi-level theoretical account of the evidence synthesized here.

The PERMA model—articulating wellbeing as comprising Positive Emotions (P), Engagement (E), Relationships (R), Meaning (M), and Accomplishment (A)—provides a directly mappable organizational structure for the PPI categories synthesized in this review. Gratitude-based interventions demonstrably target the Positive Emotions pillar, with regular gratitude practice generating reliable upward shifts in positive affect and diminished hedonic adaptation (Wood et al., 2010). Strength-based interventions operate primarily through the Engagement and Accomplishment pillars: the “use your strengths in a new way” exercise cultivates intrinsic absorption and goal-directed behavior, consistent with the flow states associated with peak engagement (Hone et al., 2015). Prosocial and loving-kindness meditation interventions most directly address the Relationships pillar, with evidence that other-directed acts generate reciprocal interpersonal warmth, social connection, and perceived social support (Aknin et al., 2013; Fredrickson et al., 2008). Meaning-centered interventions, particularly best possible self exercises and narrative strength identification, anchor wellbeing to the Meaning pillar by facilitating coherent self-narratives and a sense of purposive direction (King, 2001). Crucially, the most robustly effective PPIs—notably the three-good-things gratitude exercise and the strengths-use intervention reported by (Seligman et al. 2005)—simultaneously activate multiple PERMA pillars, suggesting that inter-pillar synergy may be a key determinant of effect magnitude and durability. This interpretation aligns with Seligman's (2011) contention that wellbeing is not a unitary construct but an emergent property of multiple mutually reinforcing positive dimensions.

Fredrickson's (2001) broaden-and-build theory provides the most parsimonious mechanistic account of how PPIs produce durable wellbeing gains rather than transient mood effects. The theory posits that positive emotions broaden momentary thought-action repertoires—expanding attentional scope, creative cognition, and behavioral flexibility—and that these broadened states progressively build lasting psychological resources including resilience, social connectedness, and cognitive flexibility. The present findings are consistent with this account: PPI-induced positive emotions do not merely shift hedonic baseline but appear to initiate upward spiraling processes wherein enhanced positive affect facilitates greater social engagement, resourceful coping, and goal pursuit, further amplifying wellbeing over time (Fredrickson et al., 2008). Mindfulness-integrated PPIs are particularly well accounted for by broaden-and-build theory, as non-judgmental attentional broadening reduces negative rumination while opening cognitive space for the recognition of positive experiences that might otherwise pass unnoticed. The observed attenuation of gratitude effects in the absence of habituated practice is also theoretically coherent within this framework: without continued behavioral activation of positive emotion, the broadening-and-building spiral decelerates, and gains regress toward baseline. This finding has direct practical implications for intervention design, suggesting that sustained PPI engagement, rather than episodic exposure, is a prerequisite for durable wellbeing gain.

Self-determination theory (SDT; Ryan and Deci, 2000) illuminates the motivational architecture underpinning PPI engagement and adherence, particularly in digital and gamified delivery contexts. SDT posits that psychological wellbeing is maximized when three basic psychological needs are satisfied: autonomy (the experience of volition and self-endorsement), competence (the experience of effectiveness and mastery), and relatedness (the experience of meaningful connection with others). The present review's finding that technology-mediated PPIs demonstrate efficacy broadly comparable to face-to-face formats, and in some cases superior engagement metrics, is explicable through SDT: well-designed digital platforms can satisfy all three basic needs simultaneously by offering participant choice over activity selection (autonomy), providing progress feedback and mastery scaffolding (competence), and facilitating social sharing or peer support features (relatedness). Conversely, the elevated dropout rates observed in self-directed digital PPI formats are theoretically attributable to failures of need satisfaction: without facilitator-provided scaffolding for competence and relatedness, autonomy alone is insufficient to sustain engagement. SDT thus provides actionable design principles for next-generation digital PPI platforms. Specifically, personalized activity recommendation, social accountability features, and competence-building feedback loops should be treated as structural requirements rather than optional enhancements.

### 4.3 Comparison with earlier systematic reviews and meta-analyses

The findings of the present review are broadly consistent with the foundational meta-analytic literature on PPIs, while extending and refining prior conclusions in several important respects. Sin and Lyubomirsky's (2009) seminal synthesis reported pooled effect sizes of *d* = 0.29 for wellbeing and *d* = 0.31 for depressive symptom reduction across 51 RCTs. The present review's observed effect size range of *d* = 0.29–0.52 across meta-analyses is largely consistent with these estimates, though the upper bound reflects the inclusion of more recently developed, digitally delivered, and gamified PPI formats that were not available at the time of Sin and Lyubomirsky's synthesis. Notably, the present review identifies a systematic relationship between intervention specificity and effect magnitude: multi-component, theoretically coherent interventions targeting specific PERMA pillars consistently outperform generic “happiness-boosting” programs, a pattern not yet articulated in early meta-analytic work.

Bolier et al.'s (2013) review of 39 RCTs similarly reported positive but modest effects (SWB: *d* = 0.34; depression: *d* = 0.20) and raised early concerns about methodological quality, including overreliance on passive waitlist controls and inadequate follow-up periods. The present review confirms and extends these concerns, documenting that only 18% of included studies reported follow-up assessments beyond 6 months, a proportion virtually unchanged from the 16% reported by Bolier et al. over a decade ago. This persistent neglect of longitudinal evaluation represents one of the most consequential unresolved methodological failures in the PPI literature. This is particularly concerning given that the broaden-and-build model predicts cumulative, time-extended benefits that short-term assessment windows are structurally incapable of capturing. Chakhssi et al. (2018) review, focused exclusively on clinical populations, demonstrated larger effect sizes than non-clinical syntheses (wellbeing: *d* = 0.40; distress: *d* = 0.26), a finding confirmed in the present review and consistent with the principle that populations with greater wellbeing deficits have more room for meaningful gain from targeted positive psychology intervention.

The present review additionally advances upon Chakhssi et al. by systematically distinguishing effect profiles across diagnostic subgroups, anxiety, depression, and chronic illness populations revealing differential responsiveness that prior aggregate analyses had obscured. Carr et al.'s (2021) synthesis of digital PPIs reported effect sizes of *d* = 0.31–0.52 that closely mirror the present review's digital PPI subgroup findings. Taken together, these comparisons suggest that the present review's findings are both externally validated by prior work and internally consistent, while offering a more granular, comprehensively scoped, and theoretically integrated characterization of the evidence base than any single prior synthesis.

### 4.4 Why certain ppis work better than others: explaining differential efficacy

The heterogeneity of effect sizes across PPI categories observed in this and prior reviews raises a fundamental question: why do some PPIs produce substantially larger and more durable wellbeing gains than others? Converging evidence from the included studies points to several explanatory mechanisms that cut across intervention type, population, and delivery context.

First, person-activity fit emerges as a robust moderator of PPI effectiveness. Lyubomirsky et al.'s (2005) sustainable happiness model predicts that wellbeing gains are maximized when the specific activities performed are congruent with the individual's personality profile, values, and motivational orientation. Empirical support for this prediction is consistent: introverted individuals respond more favorably to reflective practices such as journaling and meditation, while extraverted individuals derive greater benefit from prosocial and relatedness-focused activities (Schutte and Malouff, 2019). Gratitude interventions yield stronger effects among individuals who score lower on trait gratitude at baseline, suggesting a ceiling-effect principle wherein interventions target wellbeing deficits rather than augmenting already-high strengths. These person-activity fit effects are theoretically coherent within both SDT (autonomy-consistent activity selection maximizes intrinsic motivation) and PERMA (different pillars are differentially salient for different individuals), and they underline the importance of individualized PPI prescription over one-size-fits-all program delivery.

Second, intervention dosage and habituation matter substantially. The attenuation of gratitude letter effects in the absence of continued practice, documented across multiple included studies, demonstrates that acute positive emotion induction is insufficient for lasting change. PPIs that structure repeated behavioral engagement over multiple weeks, particularly those delivered through daily or weekly micro-practices produce more durable gains than single-session or massed-dose formats. This finding is consistent with the neuroplasticity literature suggesting that repeated activation of positive affective states is required to consolidate new neural circuits associated with wellbeing baseline elevation (Davidson and McEwen, 2012). Third, intervention specificity and theoretical coherence predict effect magnitude. PPIs grounded in explicit, testable mechanisms such as the attention-retraining account of gratitude or the strength-activation model of engagement consistently outperform generic positivity programs without clear mechanistic rationale. Multi-component PPIs that deliberately target multiple PERMA pillars simultaneously produce larger effect sizes than single-component approaches, though they present greater implementation complexity. Finally, facilitator quality and therapeutic alliance, where relevant, moderate PPI outcomes in group and clinical delivery contexts, underscoring that even well-designed PPIs require skilled implementation to realize their full potential.

### 4.5 Implications for clinical practice, education, and policy

The evidence synthesized in this review carries substantive implications for clinical practice, educational settings, and mental health policy, three domains in which the scalable delivery of evidence-based wellbeing interventions is both feasible and urgently needed.

For clinical practice, the review supports a decisive shift toward positive clinical psychology frameworks that complement traditional symptom-reduction approaches with proactive wellbeing cultivation (Wood and Tarrier, 2010). Clinicians treating depression, anxiety, and chronic illness should consider integrating structured PPIs, particularly gratitude practices, strength-based interventions, and loving-kindness meditation as adjuncts to established therapeutic modalities, given the evidence that PPIs produce wellbeing gains in clinical populations beyond those achievable through symptom management alone (Chakhssi et al., 2018). The differential efficacy data reviewed here further suggest that PPI selection in clinical contexts should be guided by systematic assessment of individual character strengths, baseline wellbeing profiles, and personal value orientations, rather than by default assignment to standardized programs. Clinicians should also be alert to the person-activity fit literature: a mismatch between a prescribed PPI and a client's motivational profile may not merely reduce effectiveness but may generate frustration or demoralization that undermines therapeutic alliance. Practically, even brief PPI components such as three-good-things journaling assigned as between-session homework can meaningfully augment existing clinical protocols at minimal resource cost.

For educational settings, the evidence supports systematic integration of PPIs into school and university curricula as universal wellbeing promotion strategies. Strength-based educational approaches, incorporating VIA character strength identification and classroom-based strengths-use exercises, have demonstrated reliable effects on student engagement, academic motivation, and school belonging (Hone et al., 2015). Mindfulness-integrated PPIs adapted for educational contexts have shown promise in reducing adolescent anxiety and improving attentional regulation, with emerging evidence that these effects translate to academic performance outcomes. Digital and gamified PPI delivery platforms are particularly well-suited to educational environments given their alignment with students' existing technology habits, their capacity for personalized feedback, and their scalability across large student populations without proportional increases in facilitator cost. Educational institutions should nonetheless resist purely technology-mediated delivery without relational scaffolding: the SDT evidence reviewed here indicates that competence support and relatedness satisfaction—typically provided through teacher or peer interaction—are essential complements to digital wellbeing tools. Wellbeing literacy programs that teach students the theoretical foundations of positive psychology, equipping them to self-select and self-administer evidence-based PPIs across the lifespan, represent a high-leverage educational investment with potential for sustained population-level wellbeing impact. For mental health policy, the review provides a scientific basis for repositioning psychological wellbeing promotion from a discretionary public health activity to a core population health priority. National mental health strategies in multiple jurisdictions have begun incorporating wellbeing indicators alongside traditional illness prevalence metrics (World Health Organization, 2022), but policy-level investment in scalable, evidence-based wellbeing interventions remains disproportionately low relative to the burden of mental ill-health. The present evidence suggests that technology-mediated PPIs represent a particularly cost-effective policy instrument: digital delivery at population scale reduces per-unit cost substantially compared to clinician-delivered alternatives, while the emerging efficacy data for mobile and gamified PPI formats suggest that acceptable engagement and meaningful wellbeing gains are achievable even in the absence of professional facilitation. Policymakers should prioritize investment in three areas: (1) large-scale trials of population-level digital PPI programs with embedded economic evaluations; (2) cross-cultural adaptation and validation of evidence-based PPIs for non-WEIRD populations, particularly in low- and middle-income countries where mental health infrastructure is most constrained; and (3) integration of wellbeing outcome metrics into routine healthcare, education, and workplace performance monitoring systems, creating the data infrastructure necessary for ongoing surveillance and program evaluation. Finally, regulatory frameworks governing AI-assisted and algorithm-personalized PPI delivery require proactive development to ensure that digital wellbeing technologies operate within ethical boundaries, protect user data, and maintain fidelity to evidence-based principles as the field rapidly evolves.

A further consideration for translating this evidence into practice is the distinction between theoretical and applied frameworks of positive psychology. The foundational work of Seligman and Csikszentmihalyi (2000) established positive psychology as a science of positive subjective experience, positive individual traits, and positive institutions, offering a broad theoretical architecture that has since been operationalized through models such as PERMA (Seligman, 2011). The PPIs synthesized in this review are most closely aligned with the positive subjective experience and positive individual traits dimensions of this framework: gratitude practices, loving-kindness meditation, and best possible self exercises cultivate positive affect and hedonic wellbeing, while strength-based interventions explicitly target the development and expression of enduring positive character traits. Prosocial and meaning-centered interventions engage the eudaimonic dimension, promoting purpose, virtue, and connectedness. Fewer reviewed interventions directly operationalize the positive institutions pillar, pointing to an underdeveloped frontier in organizational and systems-level PPI design.

Linley et al. (2006) offer a complementary practical framework that moves beyond theoretical classification to articulate how positive psychology can be embedded in applied contexts through strength identification and deployment, positive relationships, and the cultivation of meaning across life domains. Where Seligman and Csikszentmihalyi's approach provides the scientific scaffolding that legitimizes PPI development and evaluation, Linley et al.'s framework provides practitioners with an action-oriented orientation for implementing positive psychology principles in real-world settings including therapy, coaching, education, and organizational development. The implications of the present review would benefit from being understood through both lenses simultaneously: clinicians and educators should draw on the theoretical rigor of the Seligman-Csikszentmihalyi tradition to select empirically supported PPIs, while orienting their implementation approach through the practitioner-facing framework offered by Linley and colleagues, ensuring that interventions are delivered in ways that are contextually sensitive, strengths-affirming, and relationally embedded. This integration of theoretical and applied frameworks offers the most coherent pathway for translating the evidence base reviewed here into durable, scalable, and contextually responsive wellbeing practice.

### 4.6 Future directions

Future research should prioritize several underdeveloped areas. Personalized positive psychology tailoring PPI type, dose, and delivery modality to individual profiles represents a compelling emerging paradigm. Idiographic analytical approaches including ecological momentary assessment and n-of-1 designs can identify optimal intervention moments and modalities for specific individuals. The convergence of AI, natural language processing, and digital health platforms creates unprecedented opportunities for adaptive PPI delivery, provided motivational architecture is designed with attention to SDT principles (Zhang et al., 2026). The field requires substantially more longitudinal research examining whether PPI-induced wellbeing changes are durable and generalize to functional outcomes. Integration with evidence-based clinical treatments demands systematic investigation, consistent with positive clinical psychology frameworks (Wood and Tarrier, 2010). Advancing a globally relevant positive psychology requires cross-cultural research that moves beyond translation toward conceptual reconstruction of wellbeing frameworks in dialogue with diverse cultural traditions. Finally, the field must embrace open science practices including pre-registration, data sharing, and registered replication reports as standard expectations.

### 4.7 Limitations

Several limitations constrain the conclusions of this review. First, publication bias was identified across all included meta-analyses, suggesting that published effect size estimates are systematically inflated. Second, the pronounced WEIRD sampling bias limits generalizability to non-Western populations. Third, substantial heterogeneity in intervention definitions, outcome measures, follow-up periods, and control condition quality complicates cross-study synthesis. Fourth, the durability of PPI effects remains inadequately established, with only 18% of included studies reporting follow-up beyond 6 months. Fifth, this review was limited to English-language publications, potentially excluding relevant non-English literature. Future systematic reviews should address these limitations through comprehensive gray literature searches, inclusion of non-English publications, and pre-registered protocols.

### 4.8 Conclusions

The evidence base for positive psychology interventions is substantial, scientifically credible, and increasingly diverse. PPIs have consistently demonstrated positive effects on wellbeing, life satisfaction, and positive affect, with growing evidence of clinically meaningful reductions in depressive symptoms. Yet the field's methodological foundations remain imperfect. Publication bias, passive control conditions, short follow-up periods, and pronounced WEIRD sampling biases represent persistent structural limitations that temper confidence in published effect estimates. The expanding frontier of technology-mediated PPIs offers compelling opportunities for scalable, personalized, and engaging delivery of evidence-based wellbeing practices. As artificial intelligence transforms both the delivery and personalization of psychological interventions, positive psychology stands at a pivotal juncture: equipped with rich evidence base and facing a transformative technological landscape that, if navigated wisely, holds genuine promise for advancing human flourishing at scale.

## Data Availability

The original contributions presented in the study are included in the article/supplementary material, further inquiries can be directed to the corresponding author.
